# Association mapping of malting quality traits in UK spring and winter barley cultivar collections

**DOI:** 10.1007/s00122-020-03618-9

**Published:** 2020-06-06

**Authors:** Mark E. Looseley, Luke Ramsay, Hazel Bull, J. Stuart Swanston, Paul D. Shaw, Malcolm Macaulay, Allan Booth, Joanne R. Russell, Robbie Waugh, the IMPROMALT Consortium, William T. B. Thomas

**Affiliations:** 1 The James Hutton Institute, Invergowrie, Dundee, DD2 5DA Scotland, UK; 2 Present Address: Xelect Ltd, Horizon House, Abbey Walk, St Andrews, Fife, KY16 9LB Scotland, UK; 3 Plant Sciences Division, College of Life Sciences, University of Dundee, Dundee, DD1 5EH Scotland, UK; 4 Present Address: Syngenta UK Ltd., Market Stainton, Market Rasen, Lincolnshire LN8 5LJ UK

## Abstract

**Key message:**

Historical malting quality data was collated from UK national and recommended list trial data and used in a GWAS. 25 QTL were identified, with the majority from spring barley cultivar sets.

**Abstract:**

In Europe, the most economically significant use of barley is the production of malt for use in the brewing and distilling industries. As such, selection for traits related to malting quality is of great commercial interest. In order to study the genetic basis of variation for malting quality traits in UK cultivars, a historical set of trial data was collated from national and recommended list trials from the period 1988 to 2016. This data was used to estimate variety means for 20 quality related traits in 451 spring barley cultivars, and 407 winter cultivars. Genotypes for these cultivars were generated using iSelect 9k and 50k genotyping platforms, and a genome wide association scan performed to identify malting quality quantitative trait loci (QTL). 24 QTL were identified in spring barley cultivars, and 2 from the winter set. A number of these correspond to known malting quality related genes but the remainder represents novel genetic variation that is accessible to breeders for the genetic improvement of new cultivars.

**Electronic supplementary material:**

The online version of this article (10.1007/s00122-020-03618-9) contains supplementary material, which is available to authorized users.

## Introduction

Barley (*Hordeum vulgare*) is the fourth largest cereal crop as measured by worldwide annual production (faostat.fao.org). Whilst most of the global crop is used for animal feed, the most economically significant use of barley is the production of alcoholic beverages following malting. Malt production is a carefully controlled process in which mature grains are germinated, thus generating and activating a variety of amylolytic; proteolytic and cell wall degrading enzymes (Briggs 2002). Together, these modify the carbohydrate and protein composition of the grain allowing the production of fermentable sugars during mashing, and efficient alcohol production during fermentation. The physical, biological and biochemical attributes of the finished malt will determine the outcome of these downstream processes and as such are fundamental to the quality and yield of the final product. For example, the activity levels of amylolytic enzymes, produced during the malting process, can determine the levels of starch conversion (Evans et al. 2005), whilst protein degradation can affect beer flavour through the amino acid composition of the wort (He et al. 2014). Malt Nitrogen (as a percentage of the overall weight) may directly correlate with levels of enzyme activity during mashing (Swanston 1980), but will also correlate negatively with malt carbohydrate, and thus the attainable alcohol yield. Similarly, the quantity of soluble material that can be extracted from the mash and the proportion of this which is fermentable also impose constraints on alcohol yield. As such, ‘malting quality’ is a generic term that refers to a disparate set of characteristics of the malt, which together influence ease of processing, alcohol yield and flavour characteristics.

Desirable malting quality characteristics vary depending on particular brewing and distilling applications and whilst barley growers and maltsters have considerable control of these characteristics through agronomic practices and malting protocols, there is also a large amount of genetic variability between barley cultivars, despite strong selection for desirable malting characteristics in breeding programmes (Horsley et al. 1995; Molina-Cano et al. 1997; Laidig et al. 2017). Therefore, malting quality traits are a high priority for barley breeders producing new varieties aimed at the brewing and distilling market. In the UK, potential new cultivars are assessed for their value for use in brewing, malt distilling, and grain distilling by the Malting Barley Committee of the Maltsters Association of Great Britain with the better cultivars being initially given a provisional approval, generally during the first year that they are placed on the Agriculture and Horticulture Development Board (AHDB) Cereals & Oilseeds Recommended Lists. Such lines then enter commercial trials before being given a final approval or, if high yielding, moved to a feed category together with other high yielding lines that failed earlier stages of the approval process. Nevertheless, malting quality traits are complex; depending on a variety of physiological processes that occur during crop development; grain filling and ripening; and during malting itself. In addition, environmental influences (both during crop production and malting) can have significant effects on key malting quality parameters (Eagles et al. 1995; Kangor et al. 2017). The exact requirements for malting quality specification vary depending on the end use of the malt, with pure malt brewing; adjunct brewing and distilling; and malt distilling forming the major grouping of malting usage types.

Many previous studies have examined the genetic basis of malting quality traits in barley. These have included QTL mapping approaches (Marquez-Cedillo et al. 2000; Kochevenko et al. 2018), association mapping (e.g. Matthies et al. 2014; Mohammadi et al. 2015; Looseley et al. 2017; Sato et al. 2018), and microarrays (Potokina et al. 2004). Early studies (e.g. Hayes et al. 1993; Marquez-Cedillo et al. 2000) concentrated on crosses between good and poor malting quality lines or between good lines from different germplasm groups. Whilst these studies provided important information about the genetic differences responsible for variation in malting quality, they did not reflect either current breeding germplasm or breeding practice where crosses tend to be made within germplasm groups and, as such, may not have been directly relevant to crop breeding. Using panels of elite barley lines, that reflect current breeding material, in association mapping analyses can identify the loci and alleles that are being manipulated to improve malting quality as well as allowing the identification of genetic variation that is segregating in current and relevant breeding material; offering a rapid route to genetic gains in new cultivars.

One of the major constraints to conducting genetic analyses of malting quality traits is the cost of phenotyping the large numbers of lines necessary to conduct robust association mapping studies. Expensive micromalting equipment and sample throughput, coupled with complex analytical procedures and the requirement for significant quantities of grain introduce considerable costs in time and money. As such, pre-existing phenotypes collated in commercial breeding programmes, or national variety registration processes are useful data sources for genetic studies of malting quality, allowing many traits to be simultaneously analysed without the requirement costly and time-consuming phenotypic analyses. In addition, such an approach collates many independent field trials across multiple growing seasons, allowing highly robust variety means to be estimated. Previously, such data sources have been used to examine genetic gains, and correlations in grain and malting quality characters (Laidig et al. 2017); assess the potential for genomic selection for malting quality (Schmidt et al. 2016) and to conduct association mapping analyses of malting quality traits in barley. Matthies et al. (2014) identified malting quality QTL in European barley cultivars based on 25 years of micromalting data, but only considered 174 lines (representing 85 spring varieties and 89 winters. Similarly, Mohammadi et al. (2015) used micromalting data from eight US barley breeding programmes in order to map malting quality traits in 1862 barley lines, although the phenotypic data set used comprised only 4976 grain analyses in total. Looseley et al. (2017) used phenotypically contrasting variety sets in conjunction with high-density genotyping to identify QTL for diastatic power, but this study tested only 48 lines and only considered a single trait. The current study is substantially broader in scope and reports a large scale GWAS of a broad range of malting quality traits across a diverse and historical collection of UK barley cultivars comprising over 800 lines in total, and using micromalting data from thousands of historical field trials. In the UK, for a new variety to be awarded Plant Breeders Rights and added to the National List (NL) it must be demonstrated in DUS testing that it is Distinct from any other listed variety, phenotypically Uniform, and these phenotypes are transmitted to the next generation (Stable) and also that it must have value for cultivation and use (VCU) in nationwide trials co-ordinated by the Plant Variety Rights and Seeds Office. Furthermore, the Agriculture and Horticulture Development Board (AHDB) selects the best new lines based on their performance in two years of NL testing and enters them in more extended nationwide trials over 22 sites to determine whether they have any advantages over existing varieties in order to produce specific recommendations to growers (https://ahdb.org.uk/rl). These recommendations are published as the ‘recommended list’ (RL) and include Malting Barley Committee (MBC) Provisional Approval for malting based on micromalting data (http://www.ukmalt.com/protocols-and-procedures) with subsequent MBC Full Approval being dependent on satisfactory commercial performance. Historical RL data shows that the mean hot water extract of varieties that have been approved by the UK Malting barley Committee on the 2018 RL was 314.7, whereas that of the non-approved varieties was 312.9 with a pairwise least significant difference of 1.6 at the 5% level (https://cereals.ahdb.org.uk/media/1382218/Table-8-Spring-barley-AHDB-Recommended-List-2018-19.pdf). Clearly, these trials data can identify differences that are commercially significant amongst closely related germplasm and therefore represent a valuable resource. Used in conjunction with high-density genotyping, such data offers the chance to generate high resolution mapping of malting quality phenotypes in order to assess the genetic variation for malting quality that exists in elite UK barley and to identify putative gene candidates; providing the potential to improve our understanding on the mechanisms of genetic control of these commercially important characters.

The study used historic malting data from UK NL and RL trials to accurately estimate variety means for a collection of winter and spring varieties representing current and past genetic variation. This information was combined with high-density SNP genotyping to identify QTL underlying genetic variation in malting quality which were then related to known and putative candidate genes that may be responsible for these effects.

## Materials and methods

### Historical malting data

Historical malting quality data was collated from fungicide treated RL/NL trials grown between harvest years 1988 and 2016 and from field trials run over 3 years as part the AGOUEB project (Thomas et al. 2014). These data included cell wall modification traits, protein modification, diastase enzyme activity, and measures of process yield (19 quality related traits) (Table 1). On average, for the spring varieties, there were 48 RL trials per year (28 treated and 20 untreated) and 30 NL trials (16 treated and 14 untreated). For the winter crop, there was an average of 44 RL trials per year (26 treated and 18 untreated) and 26 NL trials (13 treated and 13 untreated). Varieties were present in a variable number of years and trials but were always present in at least 2 years. On average, winter varieties were present for 3.7 years, and spring varieties for 3.5. Some varieties were maintained as controls, even after completing national list trials, or removal from the recommended list. This, along with the AGOUEB trial data provided a high degree of overlap with test varieties, allowing for a comparison between varieties that were never common entries in trials. For example, cv Optic was present in spring NL/RL trials over a period of 22 years, and cv Pearl was in winter NL/RL trials for 18 years (Table S1). Data for spring and winter variety sets were analysed independently given the separate trialling and malting of the two crop types and substantial population differentiation between them (Comadran et al. 2012).

**Table 1:** Summary of the malting quality traits examined in this study

Trait	Units	Description
Germinative energy 4 ml	(%)	Proportion of seed that are germinated after 72 h following wetting with 4 ml of water
Germinative energy 5 ml	(%)	Proportion of seed that are germinated after 72 h following wetting with 5 ml of water
Germinative energy 8 ml	(%)	Proportion of seed that are germinated after 72 h following wetting with 8 ml of water
Grain nitrogen	(%)	Nitrogen composition of the grain as a percentage of the total weight (dry base)
Friability	(%)	Proportion of malt passing through a mesh under constant pressure on a friabilimeter
Homogeneity	(%)	Percentage (by weight) of malt sample retained on a 2.2 mm sieve after processing on a friabilimeter
Whole corns	(%)	Percentage (by weight) of grains retaining ¾ or more of the endosperm after processing on a friabilimeter
Diastatic power	(IoB)	Total starch converting ability of the malt
Alpha amylase	(DU)	Overall alpha amylase activity
Hot water extract (HWE)	l°/Kg	Total amount of dissolved material in a simulated mash held at 65.5 °C, measured by specific gravity
Unboiled fermentability	(%)	Proportion of wort carbohydrate that comprises fermentable sugars measured on unboiled wort
Predicted spirit yield (PSY)	(l/t dm)	Fermentable Extract (Fermentability × Hot Water Extract) × 6.06: Estimated spirit yield per tonne of malt
Malt nitrogen	(%)	Nitrogen composition of the grain as a percentage (dry base)
Total soluble nitrogen	(%)	Percentage of the grain comprising soluble nitrogen (dry base)
Soluble nitrogen ratio (SNR)	–	Ratio of soluble nitrogen to total nitrogen in the malt
Wort free amino nitrogen	(mg/l)	Level of free amino nitrogen contained in the wort
Wort viscosity	(mPa/s)	Viscosity of the wort obtained from a standard mash (450 g) measured on a viscometer
Wort β-glucan	(mg/l)	Wort β-glucan derived from an IoB (450 g) mash using a fluorimetric method
Wort Col	(EBC)	Wort colour derived from an IoB (450 g) mash and measured using standard EBC colour cards
Glycosidic nitrile (GN)	ppb	Quantity of glycosidic nitrile contained in malt extract using an enzymatic assay

Trait names are shown along with the measurement units and a brief description

In order to reduce the computational requirements of the GWAS analysis, variety means were calculated for traits in each of the variety sets (winter or spring types) rather than using individual trial means. In order to restrict the analyses to phenotypes with good data coverage, only traits with at least 2000 data points (variety, site, trial series, year combinations) were considered. A REML model was fitted to each phenotypic variate using the ‘lmer’ function of the ‘lme4′ library (Bates et al. 2015) for R (R Core Development Team 2013). This analysis comprised a random model consisting of site (nested within year), trial series, variety, the interaction between year and variety, and the interaction between site and variety. From this analysis, Best Linear Unbiased Predictors (BLUPs) were taken for each variety for each of the traits analysed and used as a phenotypic trait for subsequent association analyses.

### Germplasm

A total of 858 elite spring and winter barley genotypes representing lines that had been first entered into UK National List Trials from the early 1960s to 2015 were included in this study. This did not include the hybrid six-rowed winter barley varieties trialled during this period. The number of lines of each type that were entered into NL trials in 5-year intervals is shown in Table 2. The limited data on six-rowed varieties precluded these being analysed separately with any population differentiation within the winter germplasm being accounted for in subsequent analyses.

**Table 2:** Summary of the varieties examined in this study

NL1 year	Spring Barley		Winter Barley	
	2 row	6 row	2 row	6 row
Pre 1981	7	–	4	1
1981–1985	10	–	7	1
1986–1990	14	–	17	3
1991–1995	65	–	63	11
1996–2000	100	–	92	13
2001–2005	102	–	72	15
2006–2010	81	–	29	11
2011–2015	73	–	28	7

Varieties are summarised by seasonal growth habit, row type, and year in which they were first entered for national list trials

### Genotyping

Varieties present in the trial series were genotyped using either or both of the Illumina barley 9k iSelect chip (Comadran et al. 2012) and the more recently developed Barley 50k iSelect SNP Array (Bayer et al. 2017), according to the published protocols. Given the representation of over 6000 of the 9k iSelect markers on the 50k chip, a combined genotype file was produced by merging allele calls from these two genotyping platforms in order to minimise the proportion of missing calls. A genetic map previously described in Looseley et al. (2018) was used to relate physical map positions, taken from the 2017 cv. Morex reference assembly (Mascher et al. 2017), to genetic positions.

### GWAS

Associations between malting quality traits and genotype were tested using the compressed mixed linear model algorithm implemented in the GAPIT R package (3.0) (Wang and Zhang 2018) for R (R Core Development Team 2013). Separate association analyses were conducted for each trait for which variety effects had been estimated in each crop type. Given the variable data coverage for each trait, the exact number of varieties used for association analyses varied between traits. A minimum number of 150 lines with both genotypic and phenotypic data were required for inclusion in the analysis. Details of the number of lines included in each analysis are given in Table S1). For each analysis, an appropriate population structure using principal components was applied by setting the ‘Model.selection’ option of the analysis to ‘TRUE’. A maximum of 3 principal components was included in the analysis using this method. Significantly associated markers with an FDR adjusted p value less than 0.05 and a minor allele frequency greater than 0.1 were identified. Unique QTL were identified based on linkage-disequilibrium (LD). Within sets of significant markers for each trait, pairwise LD coefficients (LD’) were calculated. Hierarchical clustering was performed using the ‘hclust’ function (as implemented in R), using 1-LD’ as the dissimilarity matrix, and the method “average”. The resulting tree was cut at a height of 0.5 (a value chosen as a compromise between ensuring that marker sets in high LD were considered to be a single QTL effect whilst minimising the possibility of grouping unlinked markers) to identify groups of markers in LD that were considered to represent distinct QTL effects. Where there was evidence for mis-mapping of associated markers (i.e. mapping to a different chromosome or chromosome arms), these markers were not used for defining the QTL interval.

To examine historical trends in QTL frequencies, variety sets (winter and spring cultivars) were divided into 8 groups, according to the year in which they were first entered for national list trials (Table 2). Minor allele frequencies at markers representing QTL peaks were calculated within each set of varieties. To test the significance of historical trends in marker frequency, a logistic regression model was fitted to each QTL with the marker genotype at the peak marker as the response, and NL1 year as the predictor, using the ‘glm’ function as implemented in R and setting the ‘family’ argument to ‘binomial(link=’logit’)’.

#### Investigation of QTL intervals

Gene content for regions close to QTL intervals were studied using gene annotations from the 2016 version of the barley genome assembly (Mascher et al. 2017). Similarly, previously reported QTL interval associations, and potential candidate genes from published studies were placed on the physical map using BLASTn searches (Deng et al. 2007), using (https://webblast.ipk-gatersleben.de/barley_ibsc/) with default search parameters. These used published gene sequence data, probe sequences or PCR primers as the query (Table S3), and the Morex genome masked pseudomolecules (Mascher et al. 2017) as the database. For each search, the most significant match with an *E*-value less than 0.001 was chosen to locate the sequence on the physical map. Selected matches were checked based on subject chromosome and approximate genetic position (relative to mapped iSelect markers) relative to existing published locations, and in all cases were found in the expected position.

## Results

### Historical data

**Fig. 1:**
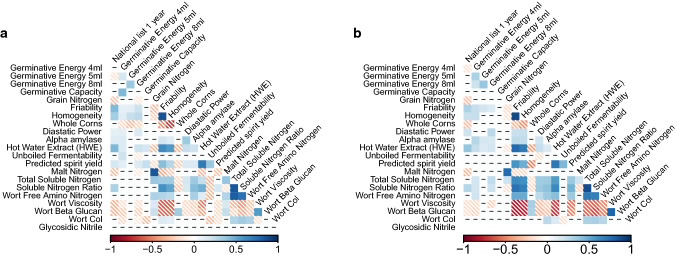
Significant correlations for BLUPs for malting quality traits examined in the current study in ** a** spring cultivars, and ** b** winter varieties. The shade corresponds to the magnitude of the correlation coefficient, with negative correlation coefficients being indicated by diagonal hatching. Where phenotypic data is not present for a pair of traits, this is indicated by a blank square

In total data from 2862 individual trials were collated, representing 1520 spring, and 1342 winter barley trials (Table 3). From this data BLUPs were calculated for 451 spring, and 407 winter varieties. These estimates were made for 26 spring malting quality traits and 25 winter traits, including grain quality, malt modification, wort attributes and process yield (Fig. 1).

**Table 3:** Summary of the trials used to derive BLUPs for each variety

	NL	RL	AGOUEB
Spring	631 (1988–2016)	854 (1988–2016)	35 (2006–2008)
Winter	519 (1988–2016)	790 (1988–2016)	33 (2006–2008)

For each seasonal habit, the number of trials from which data was collected is shown for each trial series, along with the minimum and maxim year for each of these trials

**Fig. 2:**
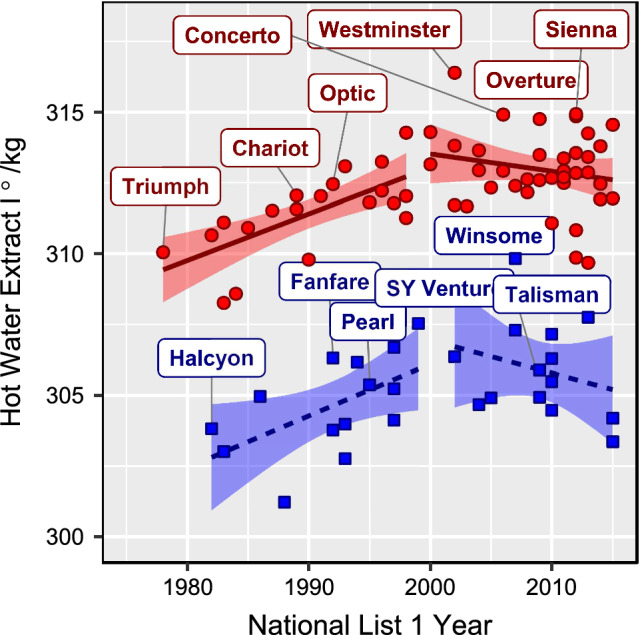
BLUPs for hot water extract plotted against year of introduction for varieties included on the recommended list and considered for malting barley committee. Spring varieties are indicated by round symbols, and winter varieties by squares, with key varieties from each set labelled. Fits from a linear regression model (and 95% CI) are shown for winter and spring sets, with separate models for varieties pre-2000 and post 2000. Fitted values for the winter set are indicated by a dashed line, and solid lines for spring models

Considering just the cultivars that had been placed on the AHDB Cereals and Oilseeds Recommended Lists and had also been considered for MBC approval, HWE BLUPs were regressed against the year in which they were first recommended to determine if genetic progress had been made. Whilst there has been highly significant genetic progress in the spring two row gene pool since the introduction of Triumph in 1980, and prior to 2000 (Fig. 2) (*β *= 0.16; *p *= 0.001; *R*^*2*^= 0.48), the rate of progress since 2000 is non-significant (*β *= − 0.06; *p *= 0.245; *R*^*2*^= 0.04) with a large scattering of the datapoints, indicating that breeding progress for the character has stalled or that the maximum attainable phenotype has been reached. The trend in the winter gene pool is less significant although the slope is similar to the springs both pre and post 2000 (*β *= 0.18; *p *= 0.034; *R*^*2*^= 0.32) and (*β *= − 0.12; *p *= 0.368; *R*^*2*^= 0.07), respectively (Fig. 2). There are, however, relatively few winter malting barley cultivars that have been released since 2000 so whilst there is no significant evidence of breeding progress, there are also too few numbers for an adequate test of genetic progress.

### Phenotypic correlations

BLUPs for the malting quality traits considered in the experiment were significantly correlated in many cases. A full correlation matrix is shown in Fig. 1. In spring barley, measures of endosperm modification were highly correlated, with friability showing a strong positive correlation with homogeneity (*r *= 0.85*, p *< 0.001) and a negative correlation with whole corns (*r *= − 0.46*, p *< 0.001). Measures of endosperm modification, particularly friability, were also correlated with hot water extract (HWE) (*r *= 0.65*, p *< 0.001) and, to a lesser extent, predicted spirit yield (PSY) (*r *= 0.41*, p *< 0.001). Germination traits generally showed low levels of correlation with other malting quality traits. Diastatic power showed moderate correlation with α-amylase activity, and both enzyme traits showed moderate levels of correlation with fermentable extract and one of its component traits (boiled fermentability). In the winter varieties, very strong positive correlations were observed between endosperm modification traits, protein modification, HWE and fermentable extract/PSY (Fig. 1). Similarly, strong negative correlations were seen between these traits and wort viscosity and beta-glucan (Fig. 1). In both sets, there was a tendency for HWE, friability and homogeneity to be positively correlated with year of introduction, and grain/malt nitrogen, wort beta glucan and wort viscosity to decrease with year of introduction (Fig. 1). The magnitude of these correlations increased when they were restricted to varieties that were marketed as malting types (Table S4). All correlations of grain and malt nitrogen with other traits were very low which may reflect the fact that the sites chosen for micromalting analyses are a subset of the whole trial set that have been selected to have a mean grain nitrogen content that would be acceptable for malting, i.e. the range of 1.4 to 1.7% grain nitrogen content. This would limit the influence of large variations in grain nitrogen content affecting malting quality parameters.

### Genotyping

From the phenotyped lines, genotypes were produced for 407 spring barley varieties and 352 winter varieties. In the overall set of spring genotypes there were 22,748 markers that had a proportion of missing values less than 0.25 and a minor allele frequency greater than 0.1. In the winters, the corresponding figure was 25,575. Because there were different numbers of genotypes that had data for each malting quality trait, the filtration was applied again for each phenotype and seasonal growth habit combination. On average, 22,275 and 25,094 SNPs were used in GWAS for the spring and winter genotypes, respectively.

### Associations

An association analysis was conducted using the BLUPs estimated from historical trial data and the derived and amalgamated 9k and 50k iSelect genotypes. This GWAS analysis identified 24 independent QTL in 13 traits in spring barley and 2 associations in 2 traits in winters (Table 4).

**Table 4:** QTL identified for malting quality traits in spring and winter barley

Set	Trait	Name	Chr	Genetic interval cM	Position Mb (interval)	Peak marker (alleles)	Minor allele (Freq)	− log10p	FDR adjusted *p* value	Minor allele effect
Spring	Homogeneity	S-HM-1	1H	13.2–13.2	0.3	JHI-Hv50k-2016-284 (G/T)	T (0.19)	3.2	0.048	− 0.45
	Diastatic power	S-DP-1	1H	2.2–2.2	1.9	SCRI_RS_124234 (C/T)	C (0.18)	4.4	0.040	3.57
	Hot water extract	S-HW-1	1H	28.2–31.8	14.2	JHI-Hv50k-2016-13067 (A/G)	G (0.41)	3.8	0.041	− 0.59
	Glycosidic nitrile	S-GN-1	1H	31.8–37.7	18.4 (14.4–18.8)	JHI-Hv50k-2016-14675 (A/G)	G (0.14)	6.3	0.002	− 108.66
	Glycosidic nitrile	S-GN-2	1H	37.7–41.9	23.1 (20–24)	JHI-Hv50k-2016-16240 (C/T)	T (0.11)	5.2	0.008	105.02
	Predicted spirit yield	S-PS-1	2H	78.7–78.7	647.3	JHI-Hv50k-2016-106390 (A/G)	G (0.23)	3.7	0.027	− 0.73
	Wort viscosity	S-WV-1	3H	1–6.9	1.5 (1.5–1.5)	JHI-Hv50k-2016-149225 (C/G)	C (0.15)	4.2	0.009	0.01
	Hot water extract	S-HW-2	3H	1–11.7	3.7 (1.7–8.9)	11_11453 (A/G)	A (0.23)	4.2	0.026	− 0.9
	Whole corns	S-WC-1	3H	27.2–27.2	22.5 (22.5–22.6)	JHI-Hv50k-2016-158667 (A/C)	C (0.1)	3.8	0.018	0.73
	Homogeneity	S-HM-1	3H	159.4–159.4	676.4	JHI-Hv50k-2016-218678 (A/G)	A (0.12)	3.6	0.023	0.62
	Friability	S-FR-1	4H	18.2–21.8	7.6 (7.6–8.7)	JHI-Hv50k-2016-228563 (A/G)	G (0.37)	3.8	0.037	− 0.96
	Friability	S-FR-2	4H	61.2–62.4	569.2 (569.2–569.5)	JHI-Hv50k-2016-256147 (A/C)	C (0.12)	3.9	0.033	− 1.42
	Homogeneity	S-HM-2	4H	62.4–62.5	569.2 (565.6–569.5)	JHI-Hv50k-2016-256147 (A/C)	C (0.12)	6.3	< 0.001	− 1.16
	Whole corns	S-WC-2	4H	60–64.5	569.8 (559.6–569.8)	JHI-Hv50k-2016-256219 (A/G)	G (0.1)	5.7	< 0.001	1.14
	Diastatic power	S-DP-2	4H	118.7–123.3	642.3 (641–645.2)	JHI-Hv50k-2016-274747 (A/G)	A (0.37)	13.5	< 0.001	− 6.57
	Germinative energy 8 ml	S-G8-1	4H	118.7–123.3	643.5 (641.8–646.2)	JHI-Hv50k-2016-275320 (A/G)	G (0.31)	6	0.004	3.66
	soluble nitrogen ratio	S-SN-1	5H	31.8–43.1	19.4	JHI-Hv50k-2016-284122 (A/G)	A (0.45)	3.9	0.046	− 0.46
	Wort viscosity	S-WV-2	5H	51.2–52.5	445 (444.2–454.3)	JHI-Hv50k-2016-304397 (A/G)	A (0.22)	3.5	0.043	0.01
	Soluble nitrogen ratio	S-SN-2	5H	83.7–87.3	532.1 (531.9–534.3)	JHI-Hv50k-2016-312337 (G/T)	T (0.25)	5.5	0.002	0.74
	Free amino nitrogen	S-FA-1	5H	83.7–88.5	532.3 (532.1–534.7)	JHI-Hv50k-2016-312374 (A/G)	A (0.27)	5.6	0.033	3.16
	Predicted spirit yield	S-PS-3	6H	68.1–68.6	478.2 (478.2–479.1)	SCRI_RS_165986 (A/C)	C (0.12)	4.3	0.014	− 0.93
	α Amylase	S-AA-1	6H	77.5–83.6	535.4 (532.8–535.8)	SCRI_RS_177093 (C/T)	C (0.13)	9.1	< 0.001	2.88
	Diastatic power	S-DP-3	6H	102.3–102.3	555.7 (554.9–555.7)	JHI-Hv50k-2016-421716 (C/G)	G (0.38)	5.3	0.006	3.6
	Whole corns	S-WC-3	7H	41.1–45.9	42.7 (41.5–45.7)	JHI-Hv50k-2016-460614 (A/T)	T (0.1)	3.7	0.023	0.69
Winter	Hot water extract	W-HW-1	1H	55.9–58.1	117 (100.5–269.2)	11_10985 (A/C)	C (0.43)	5.8	0.035	− 2.04
	Germinative energy 4 ml	W-G4-1	5H	65.6–65.6	494.3	JHI-Hv50k-2016-308754 (A/G)	G (0.49)	3.9	0.048	− 0.26

Where multiple associated SNP markers were grouped into a single QTL, a QTL interval based on the positions of the significant markers is shown

#### Endosperm modification

Two QTL were identified for friability in spring varieties. These were both located on chromosome 4H, at 7.6 Mb and 569.2 Mb. Three QTL for homogeneity were detected: on chromosome 1H at 0.3 Mb; on chromosome 3H at 676.4 Mb and on chromosome 4H at 569.2 Mb. Three QTL for whole corns were identified on chromosomes 3H, 4H and 7H. Three of the QTL for modification traits on chromosome 4H (friability at 569.2 Mb; homogeneity at 569.2 Mb and whole corns at 569.8 Mb) showed overlapping QTL intervals with minor allele effects consistent with the overall negative correlation between friability/homogeneity and whole corns.

#### Protein modification

QTL for measures of protein modification were restricted to two loci in spring barley on chromosome 5H at 19.4 Mb and 532.1 Mb. The former was associated with soluble nitrogen ratio (SNR), whilst the latter showed a highly significant association with both free amino nitrogen (FAN) (− log10p = 5.5) and SNR (− log10p = 5.6).

#### Diastase activity/germinative energy

Three QTL for diastatic power were identified in spring barley on chromosomes 1H (at 1.9 Mb); 4H (at 642.3 Mb) and 6H (at 555.7 Mb). The diastatic power QTL on chromosome 4H was collocated with a QTL for germinative energy (at 8 ml). In neither case were these associated with QTL for α-amylase activity. A single, highly significant, QTL for α-amylase activity was detected on chromosome 6H at 535.4 Mb (− log10p = 9.1). An additional QTL for germinative energy (at 4 ml) was detected in winter barley on chromosome 5H at 494.3 Mb.

#### Wort traits

Two QTL for wort viscosity were detected in spring barley at 1.5 Mb on chromosome 3H and on chromosome 5H at 445 Mb.

#### Process yield

Five QTL related to process yield were identified; four of which came from the spring barley cultivar collection. These comprised two QTL for HWE in the spring cultivar collection (on chromosome 1H at 14.2 Mb, and on 3H at 3.7 Mb) and a single QTL for HWE on chromosome 1H (at 117 Mb) in the winter cultivar collection. A single locus on chromosome 6H at 478.2 Mb had a significant effect on predicted spirit yield in the spring cultivar collection.

Two significant QTL for glyosidic nitrile (GN) were detected in the spring cultivar collection at 18.4 Mb and 23.1 Mb on chromosome 1H, although the interval of these two QTL overlapped.

### Historical trends

There was evidence for significant historical trends in allele frequencies for the majority of peak markers associated with QTL in the spring barley collection, although not for QTL identified in the winter collection (Table 5). In a number of cases, these represented large allele frequency changes over the period of time covered by the study. For example, marker 11_11453 showed a steady increase in the frequency of the marker associated with higher HWE in spring barley throughout the period considered in this study, starting as the minor allele in cultivars released before 1991 and becoming nearly fixed in the most recently released cultivars. Similarly, alleles causing an increase in diastatic power (JHI-Hv50k-2016-274747& JHI-Hv50k-2016-421716) increased substantially, becoming the major allele in recent spring barley cultivars.

**Table 5:** Frequency of the minor allele of markers corresponding to peak QTL positions identified in the current study grouped by the year in which they were first entered into UK National list trials

Marker	QTL	1963–1991	1991–1994	1994–1996	1996–1997	1997–1999	1999–2001	2001–2003	2003–2005	2005–2010	2010–2015	*p*
JHI-Hv50k-2016-284	S-HM-1	0.37	0.43	0.33	0.38	0.19	0.28	0.18	0.31	0.04	0.02	< 0.001***
SCRI_RS_124234	S-DP-1	0.17	0.22	0.26	0.08	0.16	0.23	0.15	0.33	0.12	0.09	0.19
JHI-Hv50k-2016-13067	S-HW-1	0.47	0.30	0.29	0.08	0.26	0.23	0.40	0.29	0.81	0.66	< 0.001***
JHI-Hv50k-2016-14675	S-GN-1	0.26	0.03	0.19	0.08	0.13	0.05	0.25	0.17	0.36	0.45	< 0.001***
JHI-Hv50k-2016-16240	S-GN-2	0.11	0.19	0.04	0.23	0.08	0.13	0.05	0.14	0.08	0.29	0.13
JHI-Hv50k-2016-106390	S-PS-1	0.23	0.25	0.22	0.23	0.24	0.15	0.10	0.11	0.22	0.60	< 0.001***
JHI-Hv50k-2016-149225	S-WV-1	0.63	0.45	0.30	0.23	0.21	0.10	0.15	0.03	0.03	0.02	< 0.001***
11_11453	S-HW-2	0.74	0.48	0.37	0.23	0.34	0.10	0.18	0.11	0.04	0.03	< 0.001***
JHI-Hv50k-2016-158667	S-WC-1	0.09	0.05	0.07	0.15	0.05	0.20	0.10	0.09	0.08	0.05	0.58
JHI-Hv50k-2016-218678	S-HM-1	0.11	0.21	0.12	0.23	0.11	0.21	0.13	0.06	0.13	0.09	0.35
JHI-Hv50k-2016-228563	S-FR-1	0.52	0.54	0.52	0.50	0.47	0.45	0.39	0.34	0.20	0.11	< 0.001***
JHI-Hv50k-2016-256147	S-FR-2; S-HM-2	0.34	0.23	0.15	0.38	0.24	0.08	0.08	0.06	0.07	0.05	< 0.001***
JHI-Hv50k-2016-256219	S-WC-2	0.28	0.14	0.12	0.33	0.24	0.05	0.08	0.06	0.07	0.05	< 0.001***
JHI-Hv50k-2016-274747	S-DP-2	0.54	0.43	0.52	0.46	0.50	0.53	0.50	0.33	0.22	0.11	< 0.001***
JHI-Hv50k-2016-275320	S-G8-1	0.24	0.38	0.29	0.31	0.32	0.38	0.28	0.26	0.23	0.19	0.09*
JHI-Hv50k-2016-284122	S-SN-1	0.40	0.50	0.63	0.62	0.55	0.55	0.45	0.50	0.41	0.23	0.00***
JHI-Hv50k-2016-304397	S-WV-2	0.20	0.28	0.26	0.38	0.32	0.28	0.33	0.25	0.14	0.09	0.01**
JHI-Hv50k-2016-312337	S-SN-2	0.17	0.10	0.07	0.08	0.13	0.20	0.26	0.36	0.32	0.39	< 0.001***
JHI-Hv50k-2016-312374	S-FA-1	0.14	0.08	0.11	0.08	0.13	0.18	0.25	0.31	0.30	0.40	< 0.001***
SCRI_RS_165986	S-PS-3	0.20	0.34	0.26	0.15	0.13	0.13	0.08	0.09	0.05	0.00	< 0.001***
SCRI_RS_177093	S-AA-1	0.14	0.15	0.15	0.15	0.16	0.13	0.20	0.11	0.07	0.18	0.93
JHI-Hv50k-2016-421716	S-DP-3	0.37	0.30	0.26	0.23	0.32	0.25	0.15	0.33	0.41	0.71	< 0.001***
JHI-Hv50k-2016-460614	S-WC-3	0.09	0.13	0.04	0.08	0.08	0.03	0.08	0.08	0.10	0.22	0.07*
11_10985	W-G4-1	0.63	0.52	0.58	0.50	0.64	0.56	0.40	0.27	0.50	0.71	0.97
JHI-Hv50k-2016-308754	W-HW-1	0.40	0.48	0.64	0.36	0.46	0.54	0.55	0.50	0.51	0.60	0.07*

In each case, the minor allele frequency is reported for the variety set from which the QTL was identified. Linear trends in allele frequency over time were tested using a logistic regression model

*p*-values from these tests are shown, with asterisks indicating statistical significance (***< 0.001; **< 0.01; *< 0.1)

### Investigation of QTL intervals

Where QTL effects potentially coincided with previously reported malting quality genes or QTL, published markers/sequences were related to the physical map. In total, 16 QTL/sequences from 11 studies were identified, and these are summarised in Table S3. The lack of QTL found in this study at the locations of some published major gene effects for malting quality were investigated further by checking the frequency of alleles and their trends over time for loci on the regions concerned.

## Discussion

The results generated in this study provide a summary of the genetic variation at loci influencing major malting quality parameters in current and historical UK barley cultivars. The use of data from an extremely large number of historical trials allowed robust estimates of variety means for malting quality performance across a representative set of UK growing and testing environments. Such data would have been extremely expensive and time-consuming to generate for the large number of lines necessary to run association analyses. In addition, each GWAS (spring and winter) considered around 400 varieties, allowing the identification of high confidence QTL at high resolution. In addition, the composition of the association mapping panels, representing the complete range of genetic variation in UK barley germplasm over recent decades. Information on QTL that have not become fixed in modern cultivars can be used to select for further improvement of malting quality parameters. Information on QTL that have become fixed in modern cultivars can be used in selecting appropriate parents in breeding for malting quality by ensuring that both parents contain the desirable QTL allele and relying on phenotypic selection to make further minor improvements in phenotypes.

The majority of malting quality QTL reported here were identified from spring barley. This may reflect stronger historical selection for malting quality traits in spring barley, and the incorporation (and selection of) variation that influences these traits. However, a previous study using subsets of this germplasm found substantial variation in winter varieties for a single malting quality trait (Diastatic Power) (Looseley et al. 2017), a finding not replicated here. This in turn may be due to a higher density of data for spring barley (reflecting the importance of this market in spring relative to winter barley breeding programs) in the current study, which may, provide more accurate BLUPs in this data compared to winter types, thus increasing the proportion of trait variation that can be attributed to genetic variation. Similarly, variety means were estimated for fewer winter barleys than for springs (Table S2) which will result in lower power to detect QTL. Alternatively, the relatively small number of winter malting varieties may lead to low minor allele frequencies at important malting quality loci when the entire collection is considered (unlike the study by Looseley et al. (2017), which considered equal numbers of high and low malting quality lines). In particular, current winter malting varieties are all descended from Maris Otter (Thomas et al. 2017), meaning that the genetic diversity represented in the winter malting data is much more restricted than that in the set of winter varieties overall given that only varieties aimed at the malting market will have been extensively tested for malting quality traits. The crossing and selection strategies for winter malting barley are not so focused as for the spring crop as winter malting barley is currently viewed as a declining market. The split of the winter barley market into feed and malting types is much more marked than the division in spring barley with AHDB UK recommended list winter barley trials now largely being run under a feed management regime with a small number of selected sites run under a malting management regime (https://ahdb.org.uk/knowledge-library/recommended-lists-protocols). Nevertheless, the fact that significant breeding progress has been made since the release of Halcyon (a malting variety on the UK RL from 1985 until 2000) means that there is some genetic variation to be exploited and the inability of this study to detect more QTL may well reflect the lack of power due to the far fewer numbers of lines with phenotypic data. The lack of any significant genetic progress in the crop since 2000 together with the trend towards fixation of the beneficial alleles in the more recent spring genotypes suggests that progress may well have been achieved through the inter-crossing of good malting genotypes from different NW European gene-pools and that this process had largely been completed with the release of cultivars such as Westminster (introduced 2002) and Concerto (introduced 2006). Subsequent breeding progress has been more to improve other quality aspects and/or grain yield. In the winter crop, it appears that significant progress is still being made but there are too few malting varieties released since 2000 to test this. The fact that only one QTL for malt extract was detected suggests that the optimisation of alleles already present within winter germplasm across a large number of genetic loci might be leading to the progress. Nevertheless, a narrow crossing strategy that does not lead to any new beneficial alleles being introgressed into winter barley will not be capable of narrowing the gap between winter and spring quality. A new breeding strategy is required.

**Fig. 3:**
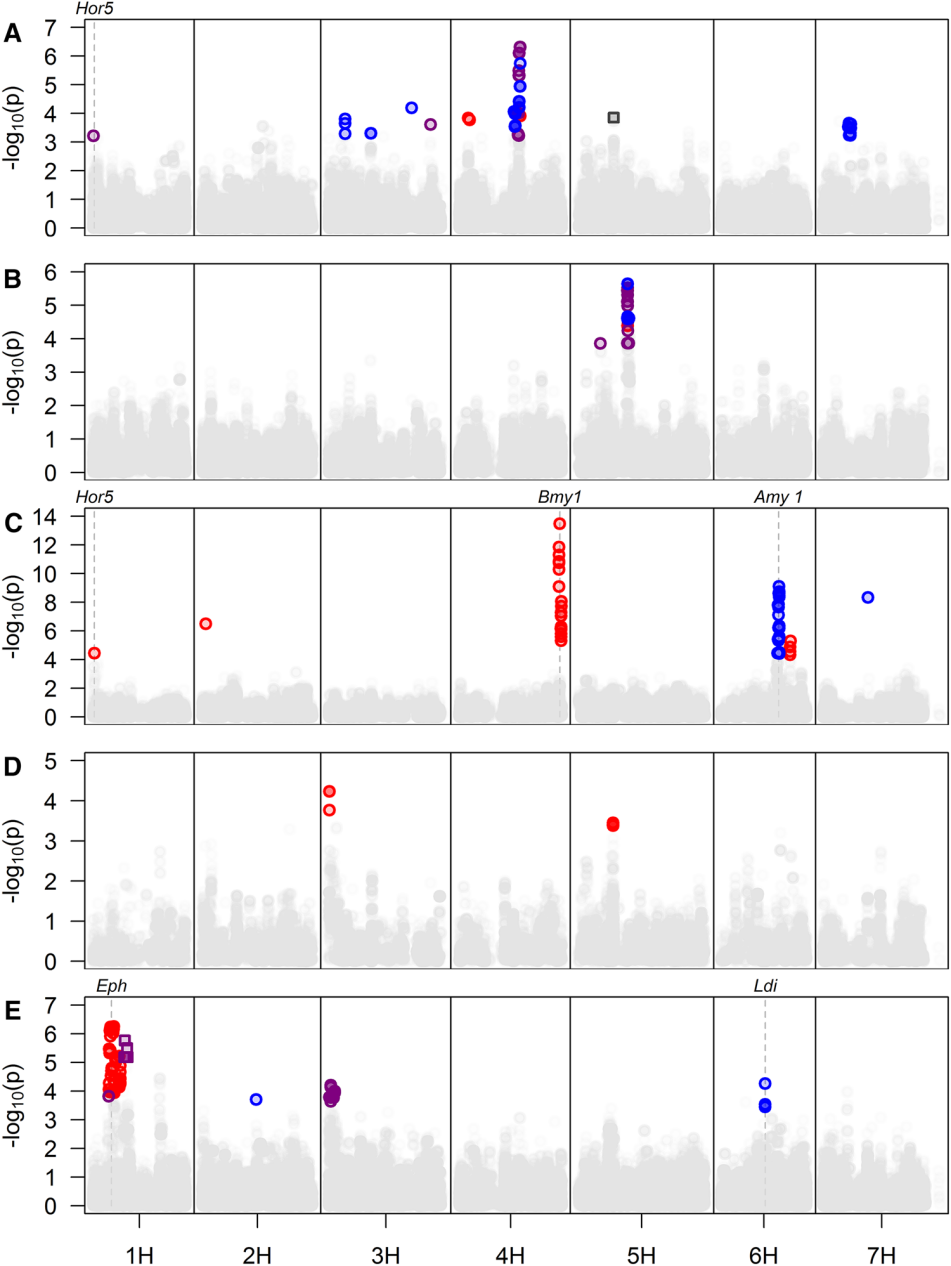
Manhatten plots summarising significant marker trait association for malting quality traits examined in this study. Multiple traits summarised in each panel, with trait groupings corresponding to those used in the main text. For each panel, the * x*-axis corresponds to a pseudo-genetic position derived by fitting physical map positions to the relationship between physical and genetic map position described in (Bayer et al. 2017). The * y*-axis corresponds to the − log10p associated with the test-statistic generated for each marker. In each pannel non-significant markers are indicated by grey symbols with significant markers shown as coloured symbols with colour and corresponding to trait, with circles relating to markers from the spring variety set, and squares the winter set. ** A** Endosperm modification: Red = Friability, Purple = Homogenetity, Blue = Whole Corns and Black = Germinative energy at 4 ml. ** B** Protien modification: Red = Total Soluble Nitrogen, Purple = Soluble Nitrogen Ratio and Blue = Wort Free Amino Nitrogen. ** C** Diastase Activity: Red = Diastatic Power and Blue = α-amylase Activity. ** D** Wort Traits: Red = Wort Viscosity. ** E** Process Yield: Red = Glycosidic Nitrile, Purple = Hot Water Extract and Blue = Predicted Spirit Yield. Names and positions of genes or loci related to specific QTL or malting quality traits are indicated by dashed lines (colour figure online)

The limitations to the variation shown for some malting quality traits by this commercial germplasm is indicated by the lack of QTL found at the location of major gene effects shown in published recent bi-parental studies (Wang et al. 2015; Kochevenko et al. 2018). Wang et al. (2015) report a major malt extract QTL on the short arm of 2H (24–35 cM peak marker GBM1121) in a DH population derived from a cross between TX9425 and Naso Nijo (a Chinese feed variety and a Japanese malting barley) and hypothesise that this due to differences at a endo-1,4-beta-xylanase (HORVU2Hr1G011550). No QTL for hot water extract or any other studied malting quality traits was found in this region in this study (Fig. 3), and the frequency of the minor allele of SCRI_RS_198643 the nearest marker to HORVU2Hr1G011550 showed no significant change over the period studied in either the spring or winter sets. Given the strength of malt extract QTL found by Wang et al. (2015) this indicates that potentially the allele in the Chinese feed barley TX9425 is not present UK commercial germplasm. Similarly, Kochevenko et al. (2018) found a major QTL hotspot effecting a number of malting traits including soluble nitrogen, friability and β-glucan on the short arm centromeric region of 3H in study of a DH population derived from two elite German spring barley lines Sofiara and Victoriana. The size of the genomic region under the peak marker in that study precluded the clear identification of a candidate gene (Kochevenko et al. 2018) or a clear comparison with the patterns of variation in the germplasm underpinning this study. However, no QTL for malting quality traits was found in this region in this study (Fig. 3) indicating that potentially the less-favourable Sofiara allele is not present UK commercial germplasm. This is potentially unexpected given the similarity of UK and German commercial germplasm but may relate to the selection of parental material exhibiting differences in drought stress tolerance in the bi-parental study (Kochevenko et al. 2018).

An analysis of correlations between BLUPs for each of the traits studied suggests that in many cases the linear relationships between malting quality traits are strong. Thus, a number of the phenotypic characters examined in this study represent similar manifestations of the same underlying malting processes. For example, both homogeneity and friability measure similar aspects of endosperm modification, are highly correlated and share a QTL position on chromosome 4H. Correlations between the year in which a variety was introduced onto the national list (NL1 year) and a number of malting quality traits are likely to reflect overall levels of historical selection. This is particularly the case in spring varieties, where NL1 year shows a high level of correlation with both HWE and fermentability, and negative correlations with nitrogen content and wort viscosity. As expected under this interpretation, the correlation coefficients for these traits are greater in absolute magnitude when restricted to malting varieties only.

Central to the malting process is the synthesis or activation of enzymes that convert starch into sugar during the malting process. The major enzymes involved in starch degradation are α-amylase, β-amylase and limit dextrinase (Evans et al. 2010). Four QTL related to diastase function were identified from spring varieties. Three QTL for diastatic power were identified on chromosome 1H at 1.9 Mb; on 4H at 642.3 Mb and on chromosome 3H at 55.7 Mb. The first of these co-locates with the position of the *Hor5* locus (encoding a γ-hordein endosperm storage polypeptide) (Shewry and Parmar 1987; Cameron-Mills and Brandt 1988). An EST (HY06A05) corresponding to a second hordein locus (*Hor2*) (Forde et al. 1985) previously associated with malting quality traits (but not diastatic power) (Potokina et al. 2004) also maps to this region (at 2.5 Mb). This result supports previous observations that have suggested that hordein concentration is associated with diastatic power (Peltonen et al. 1994), and β-amylase specifically (Wei et al. 2009), although the mechanism behind this relationship is unclear. Despite the highly significant and strong minor allele effect at this locus, the beneficial (minor) allele has decreased in frequency over the period examined in this study, although this decrease is not statistically significant. This may reflect the fact that diastatic power has not been a major breeding target in UK spring barley, or linkage drag from selection against the producer allele at the *Eph* locus, that is located close by and has been subject to recent selection (Ehlert et al. 2019).

The second diastatic power QTL was located close to the known position of *Bmy1* (Yoshigi et al. 1995) (Table S3) and collocates with a QTL reported by Looseley et al. (2017) for diastatic power in UK spring barley. β-Amylase has previously been reported to be the principle amylolytic enzyme, substantially correlating with DP (Delcour and Verschaeve 1987; Gibson et al. 1995; Santos and Riis 1996; Evans et al. 1997a, b, 2008; Georg-Kraemer et al. 2001; Li et al. 2002; Duke and Henson 2008; Duke et al. 2013). It seems likely that this QTL represents an allelic effect of the *Bmy1* locus, confirming its importance to the genetic control of diastatic power.

A single QTL for α amylase activity was identified on chromosome 6H at 535.4 Mb, with an interval of 532.8–535.8 Mb. This QTL was highly significant (− log10p = 9.1), with the minor allele causing an increase of 2.77 DU. This QTL colocalises with the known position (533.9–542.9 Mb) of a cluster of amylase genes at the *Amy1* locus (Zhang and Li 2017) and is likely to represent allelic effects or copy number variation at this locus (Mascher et al. 2017). The QTL is found in a near identical position to a QTL for α amylase activity in US barley breeding programmes (Mohammadi et al. 2015) (Table S3), although it is not clear whether the alleles identified in the current study correspond to those previously reported. Despite a previous finding showing that α-amylase activity had a positive linear relationship with diastatic power (Gibson et al. 1995) (a finding supported by a moderate positive correlation between α-amylase activity and DP in the results reported here), QTL S-AA-1 (Fig. 3) was not associated with a corresponding QTL for DP. Whilst a QTL for DP was detected on the long arm of chromosome 6H (S-DP-3; Fig. 3), this was distinct and distal to the α amylase activity QTL. Furthermore, despite the large effect associated with this locus, an analysis of allele frequency trends at the peak marker shows that the beneficial allele has remained at low frequency across the time period covered by the varieties in this study, suggesting that the allele has not been subject to positive selection in UK spring barley and may not influence primary malting quality characteristics that are under direct selection. Alternatively, there have been associations of increased α amylase levels with sprouting in the ear (Pre-Harvest Sprouting) (Lin et al. 2008) and it may be that UK breeders have avoided excessively high AA levels due to the likelihood of wet harvests leading to excessive pre-germination and malting rejections. Additionally, the beneficial allele for this locus has remained at a consistently low frequency over time suggesting that it does not influence primary malting quality characteristics that are under direct selection. Other studies have similarly concluded that α amylase activity is not the primary determinant of wort sugar in other germplasm collections (as described above) and that β-amylase is more strongly associated (Evans et al. 2008; Duke et al. 2013). Nevertheless, future genetic gains for β-amylase activity may require concurrent optimisation of other enzymes and this QTL effect represents an important determinant of α-amylase activity in current UK breeding germplasm.

Another key process in the production of malt is the modification of starchy endosperm. In order to characterise this, physical properties of malted grain are assessed in malting quality analyses through friability (overall levels of modification), homogeneity (evenness of modification) and whole corns (the proportion of wholly unmodified grain). Despite strong correlations between BLUPs for these traits, they only collocated at one QTL (S-HM-2; S-FR-2 & S-WC-4), suggesting a degree of independence in the genetic control of each of these traits. This locus on chromosome 4H has not previously been implicated in malting quality variation and does not colocalise with known malting quality genes. Nevertheless, the QTL peak is located adjacent to a gene (HORVU4Hr1G069100.2) that has high homology to a β-Xylosidase (HORVU6Hr1G075010.9) previously demonstrated to play a role in the hydrolysis of xylan oligosaccharides in barley (Lee et al. 2003) (Table S3), and which is expressed in both developing grain and embryos (https://ics.hutton.ac.uk/barleyGenes/).

In a number of cases, QTL for endosperm modification collocated with QTL for wort traits or process yield (discussed in detail below), emphasising the importance of modification to primary malt quality traits. The identification of 8 QTL related to endosperm modification in the spring cultivar set, a number of which are not fixed in current cultivars, offers significant opportunities for the optimisation of modification traits in UK cultivars.

The production of the potentially carcinogenic ethyl-carbamate during the distilling process is associated with barley varieties that produce a glycosidic nitrile known as epiheterodendrin (McGill and Morley 1990) at the *Eph* locus. This locus has been mapped to the short arm of chromosome 1H (Swanston et al. 1999) and more recently genes required for epihetrodendrin biosynthesis have been located at this locus (Knoch et al. 2016), representing a physical interval on the current genome assembly between 16.1 and 17.1 Mb on 1H. Non-production of the compound is due to a deletion of this region (Ehlert et al. 2019) and null alleles at 9k iSelect SNPs located in the deletion have been shown to be perfectly correlated with non-production of epiheterodendrin. These SNPs behave like dominant markers and are of limited value in marker-assisted selection but the recent development of a SNP assay at 17.2 Mb on this chromosome (which is highly predictive of epihetrodendrin production (https://www.huttonltd.com/services/molecular-diagnostics) solves the problem. Two distinct sets of markers (S-GN-1 & S-GN-2) associated with opposing minor allele effects on glycosidic nitrile production were identified within this region, despite the fact that the physical position of these marker sets overlapped. Whilst it is highly likely that at least one of these QTL represent an effect of alleles at the *Eph* locus, the detection of two significant but opposing effects is more likely to reflect the fact that non-production is due to the deletion. Furthermore, there is some overlap in the BLUPs estimated for non-producers compared to producers, e.g. the producer Agenda has predicted mean of 216 whereas the non-producer Corsica has a predicted mean of 255. It is likely that seasonal variations lead to imprecision in the estimation of the phenotype and thus over or under-estimation of the marker effects at individual SNP loci and these are then compensated for by detection of a QTL of opposing effect, which is most likely to be a ‘ghost’ QTL. An analysis of allele frequencies shows that the allele associated with reduced glycosidic nitrile production has increased in frequency at both QTL in spring varieties, and that this increase is highly significant. This increase coincides with the release of a molecular marker for non-producers in the early 2000s (Bringhurst 2015), illustrating the effectiveness of marker-assisted breeding in the genetic improvement of malting barley. The marker is not, however, diagnostic for non-producers of GN as it is the major allele in the elite winter barleys yet only five are non-producers. This is clearly a linked marker and its potential effectiveness in deployment for Marker Assisted Selection will depend upon the genetic background of the gene pool that a breeding programme is using.

Two QTL for hot water extract (HWE) were identified from spring variety set and one from the winter set. In the spring varieties, these were located on chromosome 1H at 14.2 Mb and on 3H at 3.7 Mb. In the winters, the HWE QTL was located on chromosome 1H at 117 Mb. This last QTL is in a (genetically) similar position to a malt extract QTL identified in a joint analysis of a European spring and winter barley cultivar collection (Matthies et al. 2014), although these QTL are located in a region of low-recombination. Whilst a number of genes with putative associations with cell wall or carbohydrate metabolism are located close to the HWE QTL peaks, little is known about the specific genetic control of malt extract traits, making the identification of candidate genes difficult. Despite the absence of shared QTL between HWE and measures of malt modification there were strong correlations between BLUPs for friability, homogeneity and HWE illustrating the importance of endosperm modification to the output of the malting process.

A locus on chromosome 6H at 478.2 Mb represented a QTL for PSY. The peak marker for this QTL (SCRI_RS_165986) was located close (in genetic distance if not physical) to the position of a gene producing a known inhibitor of limit dextrinase (LD) (Stahl et al. 2007). Variation at this locus has previously been shown to influence the activity of limit dextrinase inhibitor (Huang et al. 2014), and whilst it is not clear if the QTL represents the effect of an allele at this locus, limit dextrinase is the only enzyme capable of cleaving α-1-6 linkages in branched dextrin molecules (Manners et al. 1970). As such, the activity of the inhibitor may have a substantial effect on the fermentability of the extract. The activity of limit dextrinase inhibitor has been shown to have considerable influence on starch biosynthesis and particularly the ratio of amylose to amylopectin during grain development (Stahl et al. 2004, 2007), which may result in differential efficiency of the amylose enzymes and the capacity to reduce starch to fermentable sugars. Alternatively, the binding of limit dextrinase to its inhibitor can protect the enzyme during distillery mashing and its subsequent release during fermentation can, when coupled with alpha and beta-amylase activity, produce more fermentable sugars and hence increase spirit yield (Stenholm and Home 1999; Bringhurst et al. 2001; Walker et al. 2001). It is not known how allelic variation at the *Ldi* and/or *Ldx* loci may alter the degree to which limit dextrinase inhibitor exists in bound form, but it is a possible mechanism that may account for the observed QTL effect.

A single QTL position on chromosome 5H (532.1 Mb) was detected for wort nitrogen traits, which influenced free-amino Nitrogen and SNR, suggesting that the locus is affecting protein modification and digestion rather than absolute levels of protein. This QTL corresponds to the physical position of a marker (12_31361) previously reported to be associated with Kolbach index in a mapping population derived from two elite German spring malting barleys (Kochevenko et al. 2018). A QTL for Soluble Nitrogen has previously been reported in a similar location on chromosome 5H in a European association mapping panel, but the position of this QTL is somewhat distal to the effect reported here (Matthies et al. 2014). No gene candidate has previously been suggested for this effect, but the physical region contains a gene (HORVU5Hr1G071510) that is annotated as a Subtilisin-like protease and is expressed in early grain development and at a lower level in embryos (https://ics.hutton.ac.uk/barleyGenes/). This gene represents a strong candidate for a follow-up study. Historical allele frequency data suggest that the minor allele (increasing levels of FAN/soluble nitrogen) has increased in frequency over the period of the study but still represents the minor allele in recently released varieties. However, the requirement for protein modification will vary between brewing and distilling applications and as such strong directional selection for wort nitrogen traits is likely to be absent.

Together, these data represent an important summary of genetic variation for malting quality traits in elite UK breeding lines and, along with the associated markers, should be of considerable interest to breeders producing new varieties for the malting barley market; representing a comprehensive survey of genetic variation for malting quality in this material. In particular, the analysis of allele frequency trends will allow the selection of breeding targets that are currently segregating in elite varieties, considerably reducing the costs associated with the incorporation of novel genetic variation into existing breeding populations. In addition, by conducting a combined genomic analysis of a variety of malting quality traits, correlations between them (on an overall phenotypic level as well as at specific loci) have provided clues about their functional relationships. Candidate genes have been identified for a number of QTL, and further studies, including functional validation of these candidates, offers a route to a more complete understanding of the specific relationship between genetic variation at these loci and the physiological and biochemical processes that take place during crop development, malting, and fermentation. Such a detailed understanding of genetic relationships will provide the knowledge necessary for targeted genetic improvements in malting quality in new barley varieties.

## Electronic supplementary material

Below is the link to the electronic supplementary material.

Supplementary material 1 (XLSX 37590 kb)
